# 2-Amino-4,6-dimethyl­pyridinium benzoate

**DOI:** 10.1107/S1600536810034811

**Published:** 2010-09-04

**Authors:** Mohd Razip Asaruddin, Habibah A Wahab, Nornisah Mohamed, Mohd Mustaqim Rosli, Hoong-Kun Fun

**Affiliations:** a Pharmaceutical Design and Simulation Laboratory, School of Pharmaceutical Sciences, Universiti Sains Malaysia, 11800 USM, Penang, Malaysia; b Institute of Pharmaceutical and Neutraceuticals, Malaysia Ministry of Science and Technology and Innovation, Science Complex, 11900, Malaysia; c X-ray Crystallography Unit, School of Physics, Universiti Sains Malaysia, 11800 USM, Penang, Malaysia

## Abstract

In the title compound, C_7_H_11_N_2_^+^·C_7_H_5_O_2_^−^, the 2-amino-4,6-dimethyl­pyridinium cation and the benzoate anion are linked by two N—H⋯O hydrogen bonds, forming an *R*_2_^2^(8) ring motif. The H atoms in both the methyl groups are rotationally disordered, with fixed site occupancies of 0.50. In the crystal structure, the mol­ecules are stabilized by inter­molecular N—H⋯O hydrogen bonds. A π–π inter­action, with a centroid–centroid distance of 3.661 (2) Å, is also observed.

## Related literature

For the biological activity of Schiff bases with azomethine linkages, see Dhar & Taploo (1982 ▶). For hydrogen bonding, see: Jeffrey (1997 ▶); Jeffrey & Saenger (1991 ▶). For graph-set descriptions of hydrogen-bond ring motifs, see: Bernstein *et al.* (1995 ▶).
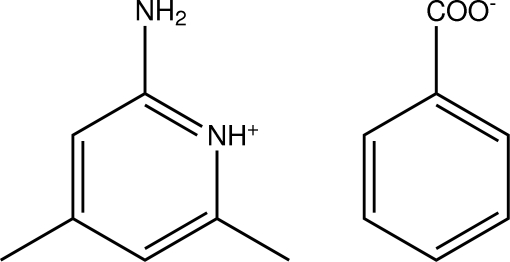


## Experimental

### 

#### Crystal data

C_7_H_11_N_2_^+^·C_7_H_5_O_2_^−^
*M* *_r_* = 244.29
Monoclinic, 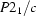

*a* = 7.5362 (16) Å
*b* = 22.937 (4) Å
*c* = 8.2124 (14) Å
β = 109.820 (2)°
*V* = 1335.5 (4) Å^3^
*Z* = 4
Mo *K*α radiation
μ = 0.08 mm^−1^
*T* = 296 K
0.57 × 0.23 × 0.05 mm

#### Data collection

Bruker SMART APEXII CCD area-detector diffractometer
Absorption correction: multi-scan (*SADABS*;p Bruker, 2009 ▶) *T*_min_ = 0.954, *T*_max_ = 0.996
7639 measured reflections
2336 independent reflections
1527 reflections with *I* > 2σ(*I*)
*R*_int_ = 0.029

#### Refinement

*R*[*F* ^2^ > 2σ(*F* ^2^)] = 0.044
*wR*(*F* ^2^) = 0.127
*S* = 1.02
2336 reflections
199 parameters
H atoms treated by a mixture of independent and constrained refinement
Δρ_max_ = 0.10 e Å^−3^
Δρ_min_ = −0.14 e Å^−3^

### 

Data collection: *APEX2* (Bruker, 2009 ▶); cell refinement: *SAINT* (Bruker, 2009 ▶); data reduction: *SAINT*; program(s) used to solve structure: *SHELXTL* (Sheldrick, 2008 ▶); program(s) used to refine structure: *SHELXTL*; molecular graphics: *SHELXTL*; software used to prepare material for publication: *SHELXTL* and *PLATON* (Spek, 2009 ▶).

## Supplementary Material

Crystal structure: contains datablocks global, I. DOI: 10.1107/S1600536810034811/fj2327sup1.cif

Structure factors: contains datablocks I. DOI: 10.1107/S1600536810034811/fj2327Isup2.hkl

Additional supplementary materials:  crystallographic information; 3D view; checkCIF report

## Figures and Tables

**Table 1:** Hydrogen-bond geometry (Å, °)

*D*—H⋯*A*	*D*—H	H⋯*A*	*D*⋯*A*	*D*—H⋯*A*
N1—H1*N*1⋯O2	1.04	1.65	2.683 (2)	172
N2—H1*N*2⋯O1	1.01	1.78	2.779 (2)	171
N2—H2*N*2⋯O1^i^	0.90	1.97	2.853 (2)	168

Symmetry code: (i) 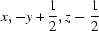.

**Fig. 1.**
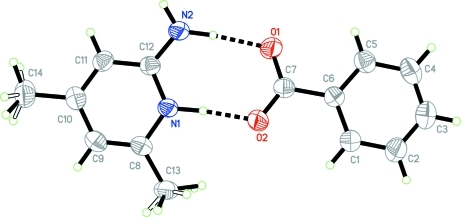
The molecular structure, showing 30% probability displacement ellipsoids and the atom-numbering scheme. Hydrogen atoms are shown as spheres of arbitrary radius. Dashed lines indicate hydrogen bonds.

**Fig. 2.**
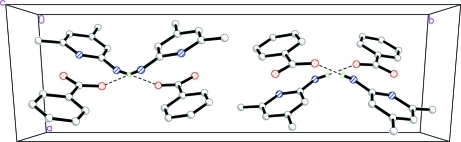
The crystal packing of (I) viewed along the c axis. Dashed lines indicate hydrogen bonds. H atoms not involved in the hydrogen bond interactions have been omitted for clarity.

## supplementary crystallographic information

### Comment

This compound is derived from 2-amino-4,6-dimethylpyridine and benzaldehyde. Schiff bases provide more potential sites for both chemical and biological activities of compounds. Schiff bases with azomethine linkage were used as anti-infectious agents (Dhar *et al.*, 1982). Pyridine and its derivatives play an important role in heterocyclic chemistry (Jeffrey, 1997). They are often involved in hydrogen-bond interactions (Jeffrey & Saenger, 1991).

The title 1:1 adduct compound contains an 2-amino-4,6-dimethylpyridinium cation and benzoate anion in the asymmetric unit. The parameters in (I), (Fig. 1), are within normal ranges. The 2-amino-4,6-dimethylpyridinium cation is planar with the maximum deviation of 0.005 (2)Å for atom C9. The H atoms of the methyl groups are disordered over two positions and with fixed site-occupancy factors of 0.50:0.50 for both of the methyl groups. The carboxylate group in benzoate anion is slightly twisted and make a dihedral angle of 7.2 (1)° with the attached benzene ring.

The 2-amino-4,6-dimethylpyridinium cation and benzoate anion groups are linked together by intermolecular N1—H1N1···O2 and N2—H1N2···O1 interactions (Table 1) forming an R^2^_2_(8) ring motif.  In the crystal structure, the molecules stabilized by intermolecular N—H···O hydrogen bonds (Table 1) and π-π interactions with Cg1—Cg2 = 3.661 (2)Å (Cg1 = N1/C8-C12, Cg2 = C1-C6).

### Experimental

An ethanol solution (20 ml) of 2-amino-4,6-dimethylpyridine (1.22 g, Aldrich) and benzaldehyde (1.06 g, Merck) were mixed, heated on a hot plate and stirred with a magnetic stirrer. The reaction mixture was refluxed for 4h. The resulting condensation solution was allowed to cool slowly at room temperature to form brownish materials. Purification was done using thin layer chromatography (TLC) and silica gel column chromatography (CC) eluted by chloroform:methanol and n-hexane:ethyl acetate solvent system. Finally the pure compound was recrystallized in ethanol which afforded the C_7_H_11_N2^+^.C_7_H_5_O_2_^-^ salt.

### Refinement

N bound H atoms was located from a difference Fourier map and were refined using a riding model, with U_iso_(H) = 1.2U_eq_(N). The methyl hydrogen atoms were located from the difference Fourier map and refined freely with the parent atom [U_iso_(H) = 1.5U_eq_(C)]. The rest of the hydrogen atoms were positioned geometrically and refined as riding model [U_iso_(H) = 1.2U_eq_(C)].

### Crystal data

**Table:**

C_7_H_11_N_2_^+^·C_7_H_5_O_2_^−^	*F*(000) = 520
*M**_r_* = 244.29	*D*_x_ = 1.215 Mg m^−^^3^
Monoclinic, *P*2_1_/*c*	Mo *K*α radiation, λ = 0.71073 Å
Hall symbol: -P 2ybc	Cell parameters from 1881 reflections
*a* = 7.5362 (16) Å	θ = 2.8–30.1°
*b* = 22.937 (4) Å	µ = 0.08 mm^−^^1^
*c* = 8.2124 (14) Å	*T* = 296 K
β = 109.820 (2)°	Plate, colourless
*V* = 1335.5 (4) Å^3^	0.57 × 0.23 × 0.05 mm
*Z* = 4	

### Data collection

**Table:**

Bruker SMART APEXII CCD area-detector diffractometer	2336 independent reflections
Radiation source: fine-focus sealed tube	1527 reflections with *I* > 2σ(*I*)
graphite	*R*_int_ = 0.029
φ and ω scans	θ_max_ = 25.0°, θ_min_ = 2.8°
Absorption correction: multi-scan (*SADABS*;p Bruker, 2009)	*h* = −8→8
*T*_min_ = 0.954, *T*_max_ = 0.996	*k* = −27→26
7639 measured reflections	*l* = −9→9

### Refinement

**Table:**

Refinement on *F*^2^	Primary atom site location: structure-invariant direct methods
Least-squares matrix: full	Secondary atom site location: difference Fourier map
*R*[*F*^2^ > 2σ(*F*^2^)] = 0.044	Hydrogen site location: inferred from neighbouring sites
*wR*(*F*^2^) = 0.127	H atoms treated by a mixture of independent and constrained refinement
*S* = 1.02	*w* = 1/[σ^2^(*F*_o_^2^) + (0.0636*P*)^2^ + 0.0715*P*] where *P* = (*F*_o_^2^ + 2*F*_c_^2^)/3
2336 reflections	(Δ/σ)_max_ < 0.001
199 parameters	Δρ_max_ = 0.10 e Å^−^^3^
0 restraints	Δρ_min_ = −0.14 e Å^−^^3^

### Special details

**Table:**

Geometry. All esds (except the esd in the dihedral angle between two l.s. planes) are estimated using the full covariance matrix. The cell esds are taken into account individually in the estimation of esds in distances, angles and torsion angles; correlations between esds in cell parameters are only used when they are defined by crystal symmetry. An approximate (isotropic) treatment of cell esds is used for estimating esds involving l.s. planes.
Refinement. Refinement of F^2^ against ALL reflections. The weighted R-factor wR and goodness of fit S are based on F^2^, conventional R-factors R are based on F, with F set to zero for negative F^2^. The threshold expression of F^2^ > 2sigma(F^2^) is used only for calculating R-factors(gt) etc. and is not relevant to the choice of reflections for refinement. R-factors based on F^2^ are statistically about twice as large as those based on F, and R- factors based on ALL data will be even larger.

### Fractional atomic coordinates and isotropic or equivalent isotropic displacement parameters (Å^2^)

**Table:**

	*x*	*y*	*z*	*U*_iso_*/*U*_eq_	Occ. (<1)
O1	0.5868 (2)	0.30973 (6)	0.23891 (18)	0.0874 (5)	
O2	0.5079 (2)	0.40213 (6)	0.18422 (17)	0.0833 (5)	
C1	0.6431 (3)	0.42956 (9)	0.5357 (3)	0.0703 (5)	
H1A	0.5807	0.4584	0.4578	0.084*	
C2	0.7115 (3)	0.44196 (11)	0.7090 (3)	0.0868 (6)	
H2A	0.6943	0.4789	0.7480	0.104*	
C3	0.8046 (3)	0.40029 (12)	0.8240 (3)	0.0924 (7)	
H3A	0.8500	0.4087	0.9418	0.111*	
C4	0.8316 (3)	0.34597 (11)	0.7670 (3)	0.0872 (7)	
H4A	0.8968	0.3177	0.8460	0.105*	
C5	0.7621 (3)	0.33309 (9)	0.5921 (3)	0.0722 (5)	
H5A	0.7807	0.2962	0.5535	0.087*	
C6	0.6651 (2)	0.37496 (8)	0.4747 (2)	0.0583 (5)	
C7	0.5808 (3)	0.36135 (8)	0.2857 (2)	0.0635 (5)	
N1	0.33910 (19)	0.38091 (6)	−0.15464 (18)	0.0574 (4)	
H1N1	0.3994	0.3858	−0.0211	0.069*	
N2	0.4633 (2)	0.28852 (6)	−0.1151 (2)	0.0734 (5)	
H1N2	0.5218	0.2955	0.0136	0.088*	
H2N2	0.4965	0.2603	−0.1752	0.088*	
C8	0.2395 (2)	0.42572 (7)	−0.2537 (2)	0.0616 (5)	
C9	0.1624 (3)	0.41807 (9)	−0.4262 (3)	0.0703 (5)	
H9A	0.0956	0.4485	−0.4947	0.084*	
C10	0.1811 (3)	0.36533 (9)	−0.5041 (2)	0.0692 (5)	
C11	0.2816 (3)	0.32162 (8)	−0.4016 (2)	0.0660 (5)	
H11A	0.2959	0.2863	−0.4511	0.079*	
C12	0.3632 (3)	0.32926 (7)	−0.2236 (2)	0.0582 (5)	
C13	0.2269 (4)	0.47999 (10)	−0.1583 (4)	0.0817 (7)	
H13A	0.086 (9)	0.490 (3)	−0.181 (9)	0.123*	0.50
H13B	0.285 (10)	0.475 (3)	−0.023 (9)	0.123*	0.50
H13C	0.298 (10)	0.513 (2)	−0.195 (8)	0.123*	0.50
H13D	0.155 (12)	0.506 (3)	−0.232 (7)	0.123*	0.50
H13E	0.344 (8)	0.494 (3)	−0.087 (10)	0.123*	0.50
H13F	0.184 (10)	0.470 (3)	−0.045 (10)	0.123*	0.50
C14	0.0940 (5)	0.3577 (2)	−0.6963 (3)	0.0984 (8)	
H14A	0.143 (11)	0.323 (5)	−0.738 (10)	0.148*	0.50
H14B	0.117 (14)	0.392 (3)	−0.758 (11)	0.148*	0.50
H14C	−0.041 (12)	0.347 (3)	−0.724 (9)	0.148*	0.50
H14D	0.071 (13)	0.319 (5)	−0.722 (11)	0.148*	0.50
H14E	0.179 (10)	0.376 (5)	−0.759 (10)	0.148*	0.50
H14F	−0.037 (11)	0.382 (3)	−0.749 (9)	0.148*	0.50

### Atomic displacement parameters (Å^2^)

**Table:**

	*U*^11^	*U*^22^	*U*^33^	*U*^12^	*U*^13^	*U*^23^
O1	0.1331 (13)	0.0527 (8)	0.0720 (9)	−0.0037 (8)	0.0288 (8)	0.0009 (7)
O2	0.1135 (12)	0.0558 (8)	0.0663 (9)	−0.0009 (7)	0.0119 (8)	0.0059 (7)
C1	0.0710 (12)	0.0710 (13)	0.0675 (13)	0.0012 (10)	0.0216 (10)	−0.0006 (10)
C2	0.0918 (16)	0.0927 (16)	0.0724 (15)	0.0031 (12)	0.0231 (12)	−0.0119 (13)
C3	0.0939 (16)	0.114 (2)	0.0648 (14)	−0.0117 (14)	0.0210 (12)	−0.0052 (14)
C4	0.0785 (14)	0.0990 (18)	0.0745 (15)	−0.0042 (12)	0.0135 (11)	0.0235 (13)
C5	0.0718 (12)	0.0666 (12)	0.0748 (14)	−0.0054 (9)	0.0204 (10)	0.0104 (10)
C6	0.0551 (10)	0.0569 (11)	0.0638 (11)	−0.0076 (8)	0.0212 (8)	0.0039 (9)
C7	0.0713 (12)	0.0520 (11)	0.0673 (12)	−0.0098 (9)	0.0238 (9)	0.0053 (10)
N1	0.0639 (9)	0.0481 (8)	0.0611 (9)	−0.0003 (7)	0.0223 (7)	0.0034 (7)
N2	0.1069 (13)	0.0532 (9)	0.0645 (10)	0.0139 (8)	0.0348 (9)	0.0070 (8)
C8	0.0552 (10)	0.0549 (11)	0.0730 (13)	0.0005 (8)	0.0192 (9)	0.0079 (9)
C9	0.0616 (11)	0.0704 (13)	0.0755 (14)	0.0094 (9)	0.0187 (10)	0.0136 (10)
C10	0.0580 (11)	0.0871 (14)	0.0641 (12)	0.0035 (10)	0.0228 (9)	0.0068 (11)
C11	0.0721 (12)	0.0667 (12)	0.0664 (12)	0.0017 (9)	0.0328 (10)	−0.0042 (9)
C12	0.0643 (11)	0.0513 (10)	0.0650 (12)	0.0000 (8)	0.0297 (9)	0.0049 (9)
C13	0.0840 (17)	0.0544 (13)	0.0937 (19)	0.0083 (11)	0.0132 (14)	−0.0013 (12)
C14	0.093 (2)	0.130 (3)	0.0671 (15)	0.014 (2)	0.0203 (14)	0.0021 (16)

### Geometric parameters (Å, °)

**Table:**

O1—C7	1.250 (2)	C8—C9	1.348 (3)
O2—C7	1.249 (2)	C8—C13	1.491 (3)
C1—C2	1.369 (3)	C9—C10	1.398 (3)
C1—C6	1.380 (3)	C9—H9A	0.9300
C1—H1A	0.9300	C10—C11	1.362 (3)
C2—C3	1.360 (3)	C10—C14	1.500 (3)
C2—H2A	0.9300	C11—C12	1.391 (3)
C3—C4	1.370 (3)	C11—H11A	0.9300
C3—H3A	0.9300	C13—H13A	1.04 (6)
C4—C5	1.384 (3)	C13—H13B	1.06 (7)
C4—H4A	0.9300	C13—H13C	1.04 (5)
C5—C6	1.381 (3)	C13—H13D	0.89 (6)
C5—H5A	0.9300	C13—H13E	0.93 (6)
C6—C7	1.497 (3)	C13—H13F	1.11 (7)
N1—C12	1.352 (2)	C14—H14A	0.99 (9)
N1—C8	1.367 (2)	C14—H14B	0.98 (10)
N1—H1N1	1.0414	C14—H14C	1.00 (8)
N2—C12	1.335 (2)	C14—H14D	0.90 (10)
N2—H1N2	1.0101	C14—H14E	1.03 (10)
N2—H2N2	0.9002	C14—H14F	1.09 (7)
			
C2—C1—C6	121.1 (2)	N1—C12—C11	118.47 (16)
C2—C1—H1A	119.4	C8—C13—H13A	109 (3)
C6—C1—H1A	119.4	C8—C13—H13B	112 (3)
C3—C2—C1	120.0 (2)	H13A—C13—H13B	104 (4)
C3—C2—H2A	120.0	C8—C13—H13C	109 (3)
C1—C2—H2A	120.0	H13A—C13—H13C	113 (4)
C2—C3—C4	120.2 (2)	H13B—C13—H13C	109 (4)
C2—C3—H3A	119.9	C8—C13—H13D	109 (4)
C4—C3—H3A	119.9	H13A—C13—H13D	52 (4)
C3—C4—C5	120.1 (2)	H13B—C13—H13D	137 (5)
C3—C4—H4A	120.0	H13C—C13—H13D	65 (4)
C5—C4—H4A	120.0	C8—C13—H13E	113 (4)
C6—C5—C4	120.0 (2)	H13A—C13—H13E	137 (4)
C6—C5—H5A	120.0	H13B—C13—H13E	54 (4)
C4—C5—H5A	120.0	H13C—C13—H13E	57 (4)
C1—C6—C5	118.58 (18)	H13D—C13—H13E	116 (5)
C1—C6—C7	120.30 (16)	C8—C13—H13F	111 (3)
C5—C6—C7	121.10 (17)	H13A—C13—H13F	68 (4)
O2—C7—O1	123.88 (17)	H13C—C13—H13F	136 (4)
O2—C7—C6	118.14 (16)	H13D—C13—H13F	115 (5)
O1—C7—C6	117.98 (16)	H13E—C13—H13F	92 (5)
C12—N1—C8	122.30 (16)	C10—C14—H14A	112 (5)
C12—N1—H1N1	117.7	C10—C14—H14B	111 (5)
C8—N1—H1N1	120.0	H14A—C14—H14B	108 (6)
C12—N2—H1N2	122.4	C10—C14—H14C	108 (4)
C12—N2—H2N2	109.7	H14A—C14—H14C	102 (7)
H1N2—N2—H2N2	125.7	H14B—C14—H14C	115 (6)
C9—C8—N1	118.79 (17)	C10—C14—H14D	110 (6)
C9—C8—C13	125.41 (18)	H14B—C14—H14D	135 (7)
N1—C8—C13	115.79 (18)	H14C—C14—H14D	67 (5)
C8—C9—C10	121.30 (17)	C10—C14—H14E	111 (4)
C8—C9—H9A	119.4	H14A—C14—H14E	77 (5)
C10—C9—H9A	119.4	H14C—C14—H14E	138 (5)
C11—C10—C9	118.37 (18)	H14D—C14—H14E	112 (7)
C11—C10—C14	121.2 (2)	C10—C14—H14F	112 (3)
C9—C10—C14	120.5 (2)	H14A—C14—H14F	132 (6)
C10—C11—C12	120.77 (18)	H14B—C14—H14F	72 (5)
C10—C11—H11A	119.6	H14C—C14—H14F	46 (4)
C12—C11—H11A	119.6	H14D—C14—H14F	109 (7)
N2—C12—N1	117.34 (16)	H14E—C14—H14F	104 (6)
N2—C12—C11	124.19 (17)		
			
C6—C1—C2—C3	0.7 (3)	C12—N1—C8—C9	0.1 (2)
C1—C2—C3—C4	0.5 (4)	C12—N1—C8—C13	−179.6 (2)
C2—C3—C4—C5	−0.8 (4)	N1—C8—C9—C10	0.7 (3)
C3—C4—C5—C6	−0.1 (3)	C13—C8—C9—C10	−179.7 (2)
C2—C1—C6—C5	−1.6 (3)	C8—C9—C10—C11	−0.8 (3)
C2—C1—C6—C7	176.91 (18)	C8—C9—C10—C14	179.9 (2)
C4—C5—C6—C1	1.3 (3)	C9—C10—C11—C12	0.2 (3)
C4—C5—C6—C7	−177.18 (18)	C14—C10—C11—C12	179.5 (2)
C1—C6—C7—O2	7.2 (3)	C8—N1—C12—N2	179.88 (16)
C5—C6—C7—O2	−174.36 (18)	C8—N1—C12—C11	−0.6 (2)
C1—C6—C7—O1	−172.47 (18)	C10—C11—C12—N2	179.94 (18)
C5—C6—C7—O1	6.0 (3)	C10—C11—C12—N1	0.5 (3)

### Hydrogen-bond geometry (Å, °)

**Table:**

*D*—H···*A*	*D*—H	H···*A*	*D*···*A*	*D*—H···*A*
N1—H1N1···O2	1.04	1.65	2.683 (2)	172
N2—H1N2···O1	1.01	1.78	2.779 (2)	171
N2—H2N2···O1^i^	0.90	1.97	2.853 (2)	168

Symmetry codes: (i) *x*, −*y*+1/2, *z*−1/2.
